# The effect of home care based on the Neuman systems model on symptomatic relief and quality of life in patients undergoing hemodialysis

**DOI:** 10.4314/ahs.v20i4.35

**Published:** 2020-12

**Authors:** Kevser Işik, Behice Erci

**Affiliations:** 1 Department of Public Health Nursing, Faculty of Health Sciences, KSÜ, Kahramanmaraş, Turkey; 2 Department of Public Health Nursing, the Faculty of Nursing, Inonu University, Malatya, Turkey

**Keywords:** Hemodialysis, dialysis symptom, quality of life

## Abstract

**Background:**

Chronic renal failure (CRF) is an important common health problem with high morbidity and mortality rate in the world and in Turkey.

**Objectives:**

This study was conducted to determine the effect of home care based on the Neuman Systems Model on relief of physical and psychological symptoms and quality of life in patients undergoing hemodialysis.

**Methods:**

This study was conducted as a pretest-posttest randomized controlled trial. The sample of the study was composed of 160 hemodialysis patients. The patients were randomly and sequentially assigned to experimental and control groups as 80 hemodialysis patients.

**Results:**

After the intervention, it was determined that the symptoms levels of the patients in the experimental group reduced and their quality of life increased.

**Conclusion:**

The care provided based on the Neuman Systems Model reduced the symptoms of the patients having hemodialysis treatment and enhanced their quality of life. Care given using a model is important in improving the quality of life of hemodialysis patients.

## Introduction

Chronic renal failure (CRF) is an important common health problem with high morbidity and mortality rate in the world and in Turkey. In America, there are approximately 23 million CRF patients.^1^ In Turkey, it has been reported that the prevalence of chronic renal failure in the population over the age of 18 is 15.7% and one out of every six adults has the chronic renal failure. Hemodialysis is the most common treatment method in the treatment of renal failure both in the world and in Turkey.^2^,^3^ In Europe, more than 180,000 patients receive hemodialysis treatment.^4^ In Turkey, the number of patients receiving hemodialysis treatment is 53.606.^5^

Lack of energy, fatigue, lack of appetite, pain, nausea, itching, shortness of breath, muscle cramps, sexual problems, and sleep disorders are the most common symptoms experienced by patients after the hemodialysis treatment. These symptoms cause poor quality of life of the patients.^6^,^7^

Especially, the duration of dialysis, the decrease in the physical functions and social relations, anxiety, unemployment, and sexual problems are the stressors that affect the patients' quality of life negatively.^6^,^8^–^10^ It has been determined in the studies that the life quality of the patients undergoing hemodialysis is low.^9^,^11^ Wyld et al (2012) found that the life quality of the patients undergoing hemodialysis were lower compared to the patients having renal transplantation.^1^

Identification of physiological, psychological and social stressors is very important in improving the quality of life and coping with hemodialysis symptoms of patients.^8^ Hemodialysis treatment affects patients in all aspects. Therefore, holistic approach is very important in nursing care.^12^ Neuman Systems Model (NSM) provides a holistic approach (physiological, psychological, sociocultural, spiritual).^13^,^14^ The model focus on stress and the reactions to stress. The use of the Neuman System Model provides nurses with the opportunity to evaluate all aspects of their patients.^14^,^15^ According to Neuman, people experience three types of stress: intrapersonal, interpersonal, and extra-personal. The Neuman System Model includes defense circles that protect the patients from the negative effects of stress. The patients can cope with stress by strengthening these defense circles.^12^,^16^

## Hypotheses

**H1:** The home care based on Neuman Systems Model reduces the severity of the physical and psychological symptoms of the patients undergoing hemodialysis.

**H2:** The home care based on Neuman Systems Model increases the life quality of patients undergoing hemodialysis.

## Methods

### Study design and sample

The study is a randomised controlled trial. The population of the study was composed of 290 adult patients receiving treatment in the hemodialysis units of three hospitals. The sample of the study was determined 160 patients (the effect size of 0.6, a power of representing population of 0.97, the significance level of 0.05). The patients were randomly assigned to experimental and control groups as 80 patients in each group. While the experimental group consisted of the patients undergoing dialysis on Monday-Wednesday-Friday, the control group consisted of the patients having dialysis treatment on Tuesday-Thursday-Saturday. The patients were listed and selected using a random numbers table. As the patients received hemodialysis treatment in different days, there was no contamination between the experimental and the control groups.

### Inclusion criteria for the study

- Being open to communication

### Exclusions criteria

- Being communication problem
- Receiving peritoneal dialysis

### Data collection

The data were collected by using the descriptive questionnaire form, dialysis symptom index and SF-36 quality of life scale.

### Descriptive Questionnaire Form

This form was composed of a total of 17 questions (gender, age, education level, marital status, number of children, profession, social security, income level, the duration of disease and treatment, another chronic disease, social support, thinking of getting rid of the disease etc.).

### Dialysis symptom index (DSI)

The Dialysis Symptom Index was developed by Weisboard et al. in 2004 to determine the hemodialysis patients' physical and emotional symptoms in the last week and the severity of these symptoms. The score received from the index, composed of 30 items, ranges between 0 and 150. The increase of the score shows that the effect of the symptom increases. Turkish validity and reliability of the Dialysis Symptom Index was conducted by Önsöz and Yeşilbalkan in 2011. The Cronbach's Alpha coefficient of the study was determined as 0.83.7 The Cronbach's Alpha coefficient of this study was determined as 0.88.

### SF-36 quality of life scale

The questionnaire developed by Ware in 1987 is composed of 36 questions and 8 sub-dimension (physical functioning, physical role, pain, general health, vitality, social functioning, mental role, mental health). The score received from the scale, ranges between 0 and 100. While 0 score indicates poor health, 100 score indicate well-being. Turkish validity and reliability of the scale was conducted by Koçyiğit et al.^17^ . The Cronbach's Alpha coefficients of the sub-dimensions were determined between 0.73 and 0.76. The Cronbach's Alpha coefficient of this study was found between 0.87 and 0.89.

### Nursing intervention

After pretest was applied to the experimental group, NANDA (North American Nursing Diagnosis Association) nursing diagnoses were created according to the neuman system model for symptoms. The nursing diagnoses were applied to the patients' homes every other week (total three times). Flexible defense line and normal defense line were strengthened with primary and secondary prevention methods (for example; health education to deal with symptoms, catheter care, psychological support etc.) The decrease in symptoms and the increase in quality of life is a result of strengthening of resistance lines. Each home care process lasted for averagely 30–60 minutes. It was determined that all 30 symptoms in the Dialysis Symptom Index were seen in patients in the experimental group. As a result of the nursing care given, it was determined that there was a decrease in the severity of the other symptoms except for 2 sexual symptoms. No intervention was applied for the patients in the control group. Pretest and posttest were performed. The control group was visited twice. After the posttest data were collected, the booklet, prepared for eliminating the symptoms and supporting the care, was delivered to the patients in the control group.

### Variables of the study

**Dependent variable:** The symptoms observed in the hemodialysis patients, quality of life

**Independent variable:** The care provided for the patients based on the Neuman Systems Model

Control variable: gender, age, education level, marital status, occupation, disease duration, duration of receiving hemodialysis treatment.

### Evaluation of data

The data obtained from the study were assessed in SPSS 17 (Statistical Package for Social Science) packaged software. The G*Power analysis that was conducted to determine the sample size of the study. Number, percentage, mean, standard deviation, independent samples t-test and paired t-test (between experimental and control groups) were used to analyze the data. The results were accepted as statistically significant in a confidence interval of 95% and on a significance level of p<0.05.

### The Ethical principles of the study

For the study, the ethical approval was received from Health Sciences Scientific Research and Publication Ethics Committee. The aim of the study was explained to the participants and they were informed that the participation was not compulsory in accordance with the “Autonomy” principle and they may withdraw from the study at any time and the data obtained from the study would be kept confidential in accordance with the principle of “Confidentiality”.

## Results

It was determined that there was no statistically significant difference between the experimental and control groups in terms of demographic variables (gender, age, educational level, marital status, employment status, duration of disease, duration of treatment). It was determined that 61.3% of the patients in the experimental group were female, 45% were illiterate, 70% were married, 96.3% were unemployed, 58.8% were housewives, the income of 63.7% of them was equal to their expense, 80% received social support, 50% received social support from his/her spouse, 63.7% thought that he/she would recover from the disease. It was determined that 53.8% of the patients in the control group were male, 56.3% were primary school graduates, 75% were married, 95% were unemployed, 51.3% were retired, the income of 63.7% of them was equal to their expense, 87.5% of them received social support, 65.7% of them received social support from his/her spouse, and 66.2% of them thought that he/she would recover from the disease (Table 1).

**Table 1:** Descriptive characteristic of experimental and control group patients

Variables	Experimental group (S (N =80)		Control group (N=80)	
	N	%	N	%
**Gender**				
Female	49	61.3	37	46.2
Male	31	38.7	43	53.8

**Education level**				
Illiterate	36	45.0	22	27.5
Primary education	36	45.0	45	56.3
Secondary education	8	10.0	13	16.2

**Marital status**				
Married	56	70.0	60	75.0
Single	24	30.0	20	25.0

**Occupation**				
Housewive	47	58.8	30	37.5
Retired	19	23.8	41	51.3
Other	14	17.4	9	11.2

**Income**				
Less income than expenses	29	36.3	29	36.3
Income equal to expenses	51	63.7	51	63.7

**Received social support**				
Yes	64	80.0	70	87.5
No	16	20.0	10	12.5

**Social supporter**				
Spouse	32	50.0	46	65.7
Daughter-Son	15	23.4	11	15.7
Other (daughter-in-law, brother, mother)	17	26.6	13	18.6

**Getting rid of the disease**				
Yes	51	63.7	53	66.2
No	29	36.3	27	33.8

	**X±SD**		**X±SD**	

**Mean age**	58.98±14.15		59.93±14.75	
**Disease duration (month)**	96.41±85.04		81.81±60.90	
**Duration of treatment (month)**	73.02±62.29		67.86±59.79	

While the mean score of the pretest Dialysis Symptom Index of the patients in the experimental group was determined as 62.26±26.52, the posttest mean score was determined as 28.52±17.67. The post-test mean scores of the patients in the experimental group decreased compared to the pretest and the difference between the two scores was statistically significant (p=0.000). In the subscales of sf-36 Quality of Life questionnaire of the patients in the experimental group, it wafound that the Physical Health total score pretest mean score was 41.93±12.34 and posttest mean score was 53.35±11.91; the Mental Health total score pretest mean score was 37.01±12.95 and posttest mean score was 50.10±9.59; the posttest mean scores were higher compared to the pretest mean scores in the total scores and the difference between the two scores was statistically significant (p=0.000) (Table 2).

**Table 2:** Comparison of pre-test-post-test dialysis symptom index and SF-36 quality of life scale of patients in experimental group

Scales	Pre-test	Post-test		Test

	X±SD	X±SD	t	p-value
**Dialysis Symptom Index**	62.26±26.52	28.52±17.67	12.49	0.000

**SF-36 Quality of Life** **Scale Subscales and total** **score**				

Physical functioning	15.51±7.43	20.87±6.40	7.12	0.000
Physical role	4.73±1.84	5.66±1.97	3.58	0.000
Pain	7.60±3.33	9.71±2.12	7.13	0.000
General health	14.08±4.24	16.83±4.12	5.75	0.000
Vitality	10.35±5.55	15.87±3.89	10.58	0.000
Social functioning	5.47±3.13	6.61±2.05	3.81	0.000
Mental role	3.45±1.07	4.27±1.49	5.160	0.000
Mental health	17.73±6.88	23.33±4.23	7.83	0.000

**Physical Health Total Score**	41.93±12.34	53.35±11.91	9.08	0.000
**Mental Health Total Score**	37.01±12.95	50.10±9.59	10.74	0.000

The pretest Dialysis Symptom Index mean score of the patients in the control group was 58.40±24.77, the posttest mean score was 59.78±19.07. It was determined that the posttest mean score of the patients increased negatively compared to the pretest mean score and the difference between the two scores was not statistically significant (p=0.49). In the subscales of Sf-36 Quality of Life Questionnaire, it was determined that the Physical Health total score pretest mean score was 40.67±11.13, the posttest mean score was 37.05±9.68 and the posttest mean scores of the patients decreased in the negative direction and the difference between the two scores was statistically significant (p<0.001). It was determined that the Mental Health total score pretest mean score was 37.41±14.21 and the posttest mean score was 35.42±11.47. It was determined that the posttest mean score of the patients decreased negatively and the difference between the two scores was statistically insignificant (p>0.05) (Table 3).

**Table 3:** Comparison of pre-test-post-test dialysis symptom index and SF-36 quaity of life scale of patients in control group

Scales	Pre-test	Post-test	Test	

	X±SD	X±SD	t	p
**Dialysis Symptom Index**	58.40±24.77	59.78±19.07	0.68	0.49

**SF-36 Quality of Life Scale** **Subscales and total score**				

Physical functioning	14.97±7.27	13.68±5.43	2.40	0.01
Physical role	4.57±1.38	4.16±0.77	2.70	0.00
Pain	7.57±3.25	6.68±2.75	3.86	0.00
General health	13.55±2.96	12.51±4.66	1.73	0.08
Vitality	10.71±5.80	10.16±4.26	1.12	0.26
Social functioning	5.23±3.31	4.37±2.49	3.60	0.00
Mental role	3.48±1.11	3.12±0.58	3.33	0.00
Mental health	17.97±6.48	17.76±6.21	0.33	0.73

**Physical Health Total Score**	40.67±11.13	37.05±9.68	4.12	0.00

**Mental Health Total Score**	37.41±14.21	35.42±11.47	1.68	0.09

It was found that there was no statistically significant difference between the groups in terms of the Dialysis Symptom Index and sf-36 Quality of Life Questionnaire subscale pretest mean scores of the patients in the experimental and control groups (p>0.05). There was a decrease in the symptoms experienced by the patients in the experimental group after the intervention and therefore there was a statistically significant difference between the groups in terms of the Dialysis Symptom Index posttest mean scores of the patients in the experimental and control groups (p<0.001). It was determined that there was an increase in a positive way in the quality of life questionnaire posttest mean scores of the patients in the experimental group after the intervention and therefore there was a statistically significant difference between the groups in terms of posttest mean scores of sf-36 Quality of Life Questionnaire subscales of the patients in the experimental and control groups (p<0.05) (Table 4).

**Table 4:** Comparison of pre-test-post-test dialysis symptom index and SF-36 quality of life scale of patients in experimental and control groups

**Scales**	**Experimental** **Group**	**Control Group**	**Test**	

	**Pre test**	**Pre test**		
	**X±SS**	**X±SS**	**t**	**p-value**
**Dialysis Symptom Index**	62.26±26.52	58.40±24.77	0.95	0.34

**Total score of SF-36** **Quality of Life Scale**				
Physical Health	41.93±12.34	40.67±11.13	0.67	0.49
Mental Health	37.01±12.95	37.41±14.21	0.18	0.85

**Scales**	**Experimental** **Group**	**Control Group**	**Test**	
		
	**Post test**	**Post test**		
	**X±SD**	**X±SD**	**t**	**p-value**

**Dialysis Symptom Index**	28.52±17.67	59.78±19.07	10.75	0.000

**Total score of SF-36** **Quality of Life Scale**				
Physical Health	53.35±11.91	37.05±9.86	9.42	0.000
Mental Health	50.10±9.59	35.42±11.47	8.77	0.000

## Discussion

The patient having hemodialysis treatment experience lots of symptoms and these symptoms affect their quality of life negatively. The effect of the home care based on the Neuman Systems Model on the relief of symptoms and quality of life in the patients undergoing hemodialysis was discussed in this section.

In the study, it was determined that the symptoms of the patients in the experimental group decreased in the posttest based on the Dialysis Symptom Index and the difference between the pretest and posttest scores was statistically significant (p<0.001) (Table 2). The patients receiving hemodialysis treatment have many physical, psychological and social problems and these problems increase the symptoms of the patients and affect their life negatively.^7^,^18^ In the study, there was a significant decrease in the dialysis symptom index posttest scores of the patients in the experimental group. It was thought that there was a decrease in the symptoms as the Neuman Systems Model used as a guideline in the care provided an individual-centered care, made it easier to collect detailed data by evaluating the patients with a holistic view, activated the coping mechanism of the individual against the stressors caused by the disease, and strengthened the defense lines against all the negative effects of the disease. It was determined that the life quality of the patients in the experimental group increased, the posttest mean scores in the sf-36 Quality of Life Questionnaire subscales increased positively compared to pretest mean scores and the difference between the pretest and posttest scores was statistically significant (p<0.05) (Table 2). In the study by Bahadori et al., it was found that the care provided using the self-care model enhanced the quality of life of the patients undergoing hemodialysis.^19^ In the study, it was found that the symptoms of the patients in the control group increased and the difference between the pretest and posttest mean scores was statistically insignificant (p>0.05) (Table 3). As no intervention was applied to the patients in the control group, there was no decrease in the symptoms. It was determined that the posttest mean score obtained by the patients in the control group from sf-36 Quality of Life Questionnaire subscales decreased negatively compared to the pretest mean score and the difference between the pretest and posttest mean scores was statistically significant (p<0.05) (Table 3). It was determined that the significant difference was associated with the negative decrease of the mean scores in the posttest. In the study by Edraki et al. it was determined that the life quality of the individuals in the control group, for whom no intervention was applied, decreased negatively and the difference between the pretest and posttest mean scores was statistically significant.^20^

It was determined that there was no statistically significant difference between the pretest mean scores of the patients in the experimental and control groups in the Dialysis Symptom Index and there was a statistically significant difference between the posttest mean scores (p:0.000) (Table 4). The fact that there was a decrease in the Dialysis symptoms of the patients in the experimental group after the nursing care provided at home based on the Neuman Systems Model revealed that the care was effective. This result confirms the hypothesis “The home care based on Neuman Systems Model reduces the severity of the physical and psychological symptoms of the patients undergoing hemodialysis”. It was determined that while there was no statistically significant difference between the pretest mean scores of the patients in the experimental and control groups in the sf-36 Quality of Life Questionnaire, there was a statistically significant difference between the posttest mean scores (p<0.05) (Table 4). In this study, the fact that the life quality of the patients in the experimental group increased in all the subscales after the nursing care provided at home based on the Neuman Systems Model showed that the care was effective. This result confirms the hypothesis “The home care based on Neuman Systems Model increases the life quality of patients undergoing hemodialysis ”.

## Limitations

The researcher had difficulty finding the address of the patient's home.

## Conclusion

It was determined that the symptoms of the patients in the experimental group decreased and their quality of life enhanced and the symptoms of the patients in the control group increased and their quality of life impaired after the care provided based on the Neuman Systems Model. In the light of these results, as using the Neuman Systems Model as a guideline in the nursing care to be provided for the patients receiving hemodialysis treatment and providing the care based on a model affect the results positively, it is recommended for the nursing care to be provided for the patients receiving hemodialysis treatment by using different models. In the light of these results, as using the Neuman Systems Model as a guideline in the nursing care to be provided for thepatients receiving hemodialysis treatment and providing the care based on a model affect the results positively, it is recommended for the nursing care to be provided for the patients receiving hemodialysis treatment by using different models.

## References

[1] Wyld M, Morton RL, Hayen A, Howard K, Webster AC (2012). A systematic review and meta-analysis of utility-based quality of life in chronic kidney disease treatments. PLoS Medicine.

[2] Ovayolu N, Ovayolu O (2016). Basic internal diseases nursing and chronic diseases with different dimensions.

[3] Ören B, Enç N (2013). Quality of life in chronic haemodialysis and peritoneal dialysis patients in Turkey and related factors. International Journal of Nursing Practice.

[4] Parra E, Arenas MD, Alonso M, Martínez MF, Gamen Á, Aguarón J (2017). Assessing value-based health care delivery for haemodialysis. Journal of Evaluation in Clinical Practice.

[5] Ministry of Health (2016). End Stage Renal Failure-Dialysis.

[6] Mor MK, Sevick MA, Shields AM, Green JA, Palevsky PM, Arnold RM (2014). Sexual function, activity, and satisfaction among women receiving maintenance hemodialysis. Clinical Journal of the American Society of Nephrology.

[7] Önsoz HB, Usta Yesilbalkan O (2013). Reliability and validity of the turkish version of the dialysis symptom index in chronic hemodialysis patients. Turk Neph Dial Transpl.

[8] Akın S, Taşköprü İ, Özdilli K, Yeşiltepe G, Öztürk B, Durna Z (2010). The Functional Performance Status, Quality of Life and Hemodialysis Stressors of Hemodialysis Patients. Journal of Nursing Education and Research.

[9] Cavalcante MCV, Lamy ZC, Lamy Filho F, França AKTDC, Santos AMD, Thomaz EBAF (2013). Factors associated with the quality of life of adults subjected to hemodialysis in a city in northeast Brazil. Brazilian Journal of Nephrology.

[10] Yang F, Griva K, Lau T, Vathsala A, Lee E, Ng HJ (2015). Health-related quality of life of Asian patients with end-stage renal disease (ESRD) in Singapore. Quality of Life Research.

[11] Mandoorah QM, Shaheen FA, Mandoorah SM, Bawazir SA, Alshohaib SS (2014). Impact of demographic and comorbid conditions on quality of life of hemodialysis patients: a cross-sectional study. Saudi Journal of Kidney Diseases and Transplantation.

[12] Özer S, Gökçe S (2015). Applying the neuman systems model in a hemodialysis treatment case. Journal of Nursing Education and Research.

[13] Güner Ö, Kavlak O (2015). Care patient with endometrial cancer according to neuman systems model: a case report. Balıkesir Journal of Health Sciences.

[14] Pektekin Ç (2013). Nursing philosophy theories-care models and political approaches.

[15] Dağ H, Kavlak O, Şirin A (2014). Newman Systems Model and Infertility Stressors. Turkiye Klinikleri Journal of Nursing Sciences.

[16] Uysal N, Khorshid L, Eşer I (2009). A Case Study Based on Betty Neuman Systems Model. Journal of Anatolia Nursing and Health Sciences.

[17] Koçyiğit H, Aydemir Ö, Fişek G, Ölmez N, Memiş A (1999). Reliability and Validity of the Turkish Version of Short Form-36 (SF-36). İlaç ve Tedavi Dergisi.

[18] Akdemir N, Birol L (2005). Internal diseases and nursing care.

[19] Lii YC, Tsay SL, Wang TJ (2007). Group intervention to improve quality of life in haemodialysis patients. Journal of Clinical Nursing.

[20] Edraki M, Kamali M, Beheshtipour N, Amoozgar H, Zare N, Montaseri S (2014). The effect of educational program on the quality of life and self-efficacy of the mothers of the infants with congenital heart disease: a randomized controlled trial. International Journal Of Community Based Nursing And Midwifery.

